# External Validation of a Clinical Score for Patients With Neuroendocrine Tumors Under Consideration for Peptide Receptor Radionuclide Therapy

**DOI:** 10.1001/jamanetworkopen.2021.44170

**Published:** 2022-01-19

**Authors:** Satya Das, Aman Chauhan, Liping Du, Katharine E. Thomas, Aasems Jacob, Aimee Schad, Shikha Jain, Aaron Jessop, Chirayu Shah, David Eisner, Dana B. Cardin, Kristen K. Ciombor, Laura W. Goff, Marques Bradshaw, Dominique Delbeke, Martin Sandler, Robert A. Ramirez, Jordan Berlin

**Affiliations:** 1 Division of Hematology and Oncology, Department of Medicine, Vanderbilt University Medical Center, Nashville, Tennessee; 2 University of Kentucky Markey Cancer Center, Lexington; 3 Department of Biostatistics, Vanderbilt University of Medical Center, Nashville, Tennessee; 4 Division of Hematology and Medical Oncology, Department of Medicine, Lousiana State University, New Orleans; 5 Department of Medicine, Rush Medical Center, Chicago, Illinois; 6 Department of Medicine, University of Illinois Chicago, Chicago; 7 Department of Radiology, Vanderbilt University Medical Center, Nashville, Tennessee

## Abstract

**Importance:**

Despite the benefit of peptide receptor radionuclide therapy (PRRT) for patients with well-differentiated neuroendocrine tumors (WD NETs), no clinical metric to anticipate benefit from the therapy for individual patients has been previously defined.

**Objective:**

To assess whether the prognostic ability of the clinical score (CS) could be validated in an external cohort of patients with WD NETs.

**Design, Setting, and Participants:**

This multicenter cohort study’s analysis included patients with WD NETs who were under consideration for peptide receptor radionuclide therapy (PRRT) with lutetium-177 (^177^Lu)-dotatate between March 1, 2016, and March 17, 2020. The original cohort included patients from Vanderbilt-Ingram Cancer Center. The validation cohort included patients from Ochsner Medical Center, Markey Cancer Center, and Rush Medical Center. Patients with paragangliomas, pheochromocytomas and neuroblastomas were excluded. Statistical analysis was performed from June to November 2021.

**Exposures:**

PRRT with ^177^Lu-dotatate or alternate therapies such as everolimus, sunitinib, or capecitabine plus temozolomide.

**Main Outcomes and Measures:**

The primary outcome was progression-free survival (PFS) and was estimated by the Kaplan-Meier method; a Cox proportional-hazards model adjusting for primary tumor site, tumor grade, and number of PRRT doses administered was used to analyze association between CS and outcomes.

**Results:**

A total of 126 patients (median age [IQR] age: 63.6 [52.9-70.7] years; 64 male individuals) were included in the validation cohort, and the combined cohort (validation and original cohorts combined) had a total of 248 patients (median [IQR] patient age: 63.3 [53.3-70.3] years; 126 male individuals). In the validation cohort, on multivariable analysis, for each 2-point increase in CS, PFS decreased significantly (hazard ratio, 2.61; 95% CI, 1.64-4.16). After finding an association of the CS with PFS in the validation cohort, the original and validation cohorts were combined into the cohort for this analysis. On multivariable analysis, for each 2-point increase in CS, PFS decreased significantly (hazard ratio, 2.52; 95% CI, 1.89-3.36).

**Conclusions and Relevance:**

Increases in CS were associated with worsening PFS in the validation cohort, validating findings from the original cohort. These findings suggest that the CS, to our knowledge, represents the first clinical metric to estimate anticipated benefit from PRRT for patients with WD NETs and may be a clinical tool for patients being considered for PRRT.

## Key Points

**Question:**

How does a previously prospectively derived clinical score for patients with neuroendocrine tumors under consideration for peptide receptor radionuclide therapy perform prognostically in an external validation cohort?

**Findings:**

In this cohort study of 248 patients in the combined cohort, for each 2-point increase in clinical score, patients were 2.5 times as likely to experience disease progression or death.

**Meaning:**

These findings suggest that the clinical score is a validated clinical metric that can estimate the anticipated benefit from peptide receptor radionuclide therapy for individual patients with well-differentiated neuroendocrine tumors.

## Teaser

This cohort study examines a clinical score’s ability to estimate the benefit from peptide receptor radionuclide therapy for individual patients with well-differentiated neuroendocrine tumors.

## Introduction

Peptide receptor radionuclide therapy (PRRT) with lutetium-177 (^177^Lu)-dotatate has now garnered regulatory licensure in Europe and the United States for the treatment of somatostatin receptor–positive gastroenteropancreatic neuroendocrine tumors (NETs) and has demonstrated antitumor activity in somatostatin receptor positive NETs from other primary sites.^1,2,3^ Despite the benefit from ^177^Lu-dotatate, questions remain about when to sequence the therapy for patients in relation to other available therapies, and which patients are optimal candidates for the therapy. The prospective studies comparing PRRT with targeted agents are years away from results being available and still may not define the optimal time point to administer the therapy for a given patient.^4^ PRRT may also be associated with unacceptable toxicity for patients with disease involving certain sites or baseline comorbidities.^5,6,7,8^ To date, no prognostic scoring system has been developed to anticipate patient benefit from PRRT. We prospectively developed a clinical score (CS) for patients with well-differentiated (WD) NETs under consideration for ^177^Lu-dotatate to serve as such a scoring system. In our original analysis, we found the CS to be associated with progression-free survival (PFS) in patients treated with ^177^Lu-dotatate.^9^ In this manuscript, we present our efforts to validate the CS and establish it as the first clinical metric that can estimate the anticipated benefit from ^177^Lu-dotatate for patients.

## Methods

This cohort study followed the Strengthening the Reporting of Observational Studies in Epidemiology (STROBE) reporting guideline. Prior to data collection, institutional review board approval from each participating institution was obtained. The institutional review board of each participating institution granted a waiver of consent given the deidentified nature of the patient data collected for the analysis.

### Patients

The original cohort included 122 patients with WD NETs from Vanderbilt Ingram Cancer Center under consideration for ^177^Lu-dotatate between March 1, 2016, and March 17, 2020. The validation cohort included 126 patients with WD NETs from Ochsner Medical Center (n = 51), Markey Cancer Center (n = 51) and Rush Medical Center (n = 24) under consideration for ^177^Lu-dotatate between January /25, 2017, and March 6, 2020. The last follow-up date for survival assessment was December /16, 2020. All patients in the original cohort were assigned a CS prospectively while the patients in the validation cohort were retrospectively assigned a CS, with the CS-assigning investigator blinded to patient outcomes. Each patient in the analysis was deemed to be a possible PRRT candidate based upon institutional multidisciplinary tumor board review and, at minimum, possessed adequate tumor somatostatin receptor expression and hematologic reserve (all parameters defined as per inclusion criteria from the NETTER-1 trial^1^). Patients who received 0 doses of PRRT received an alternate systemic therapy due to logistical (eg, wait times and travel limitations) rather than disease-related reasons. Patients with paragangliomas, pheochromocytomas, and neuroblastomas were not included in this analysis given that non-PRRT treatment options for these tumors differ from the treatment alternatives to PRRT available for the NETs included in the study. We did not collect race and ethnicity data because they have not been demonstrated as factors influencing PRRT responsiveness.

### Data Collection

A Redcap database designed at Vanderbilt was used to generate a data collection instrument for standardized data collection. Four individual physician investigators (S.D., K.T., A.J., and A.S.), one from each site, collected data using this instrument. One investigator (S.D.) reviewed all patient data and any inconsistencies were resolved by consensus. For purposes of the analysis, grade 1 tumors were considered typical lung NETs, and grade 2 tumors were considered atypical lung NETs. Tumors that did not have a grade listed on pathology reports, nor features that would suggest grade, were characterized as low grade not otherwise specified (NOS). All features used to determine tumor grade and differentiation were gleaned from institutional pathology reports.

### Outcome Measures

**Figure 1.**
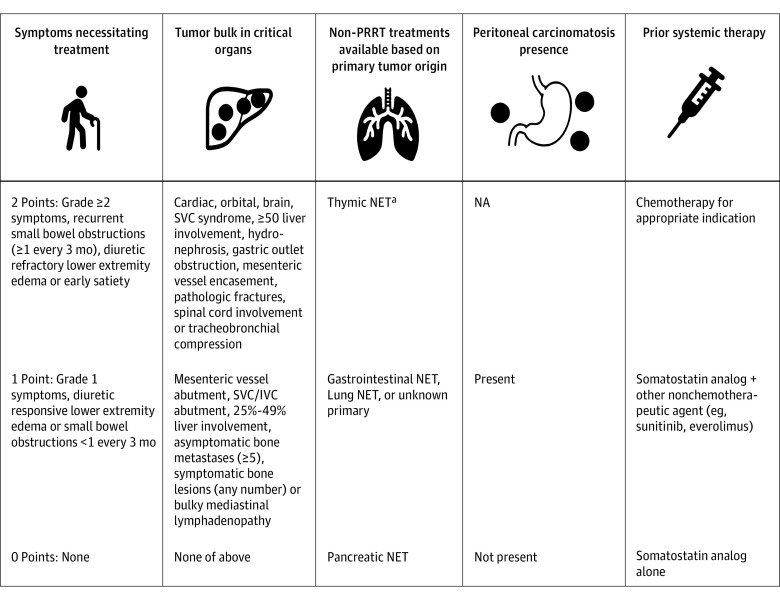
A Description of the Clinical Score and the Scoring Criteria (0, 1, 2) for Each Category Included in the Clinical Score IVC indicates inferior vena cava; NA, not applicable; NET, neuroendocrine tumor; PRRT, peptide receptor radionuclide therapy; and SVC, superior vena cava. ^a^The rationale behind the scoring criteria for this category is that patients with pancreatic NETs possess the greatest number of treatment options beyond PRRT, relative to patients with gastrointestinal or lung NETs. Patients with thymic NETs, on the other hand, possess the lowest number of treatment options beyond PRRT, relative to patients with gastrointestinal or lung NETs.

One investigator (S.D.) generated a CS for each patient based on the scoring system detailed in Figure 1 (the methodology for the CS is detailed in the original manuscript^9^). Although the validation cohort patients were scored retrospectively, the CS of these patients were generated in an outcome-blinded manner. Independent radiologists at each institution measured post-PRRT response for patients by RECIST 1.1 on cross-sectional imaging; these radiologists were unaware of patient CS during response assessment. Response outcomes were assessed from computed tomography (CT) or magnetic resonance imaging (MRI) scans and not somatostatin receptor-based imaging. Patients typically underwent initial restaging scans 1-month post-PRRT completion and thereafter, according to tumor grade (eg, every 3 months for patients with grade 2/3 tumors and every 6 months for patients with grade 1 tumors). The primary outcome of the analysis was PFS, which was defined as the time from the initial ^177^Lu-dotatate or other alternate treatment dose to date of disease progression or death. Patients who did not experience disease progression or death were censored at the date of their last clinic visit. Several secondary endpoints such as overall survival (OS), objective response rate (ORR), symptomatic benefit and development of grade 3/4 adverse events (AEs) were also assessed. OS was defined as the time between initial ^177^Lu-dotatate or other alternate treatment dose and date of death. ORR was defined as the proportion of patients who achieved complete response or partial response on any post-PRRT CT or MRI scans by RECIST 1.1. AEs were graded by Common Terminology Criteria for Adverse Events Version 5.0 and tabulated for patients who received at least one dose of ^177^Lu-dotatate.

### Statistical Analysis

Patient demographics and characteristics at baseline were summarized using descriptive statistics such as medians, lower and upper quartiles for continuous variables, and frequencies and relative frequencies for categorical variables. The Wilcoxon rank-sum test and Pearson χ^2^ test were applied when comparing a continuous variable and a categorical variable, respectively, between 2 groups. Survival times were estimated using the Kaplan-Meier method and the log-rank test was used to compare the difference between groups. To evaluate the association of the CS and PFS, a multivariable Cox regression with robust standard errors was performed adjusting for tumor grade (grade 1, grade 2/3, and low-grade NOS), primary tumor site (small intestine, pancreas, and other) and PRRT doses received (0, 1-2, 3-4). The interaction between PRRT dose and CS was tested and then removed from the model because little evidence was observed. To evaluate the possible influence of the CS on OS, a Cox regression model adjusting for PRRT doses received only, due to limited number of death events, was performed. After finding that the CS was also associated with PFS and OS in the validation cohort, we combined the validation and original cohorts into a combined cohort for this analysis to increase the estimation precision of the preceding Cox regression models. All statistical tests were 2-sided and *P *≤ .05 was considered as statistically significant. All statistical analyses were performed via R software version 4.1.1 (R Project for Statistical Computing) from June to November 2021.

## Results

### Baseline Patient Characteristics

The validation cohort included 126 patients; 64 were male, median (IQR) patient age was 63.6 (52.9-70.7) years, median (IQR) CS was 3 (3-5) points and median (IQR) duration of follow-up was 20.2 (12.4-27.2) months. From the validation cohort, on multivariable Cox regression, for each 2-point increase in CS, the hazard ratio (HR) for PFS was 2.61 (95% CI, 1.64-4.16). On multivariable Cox regression, for each 2-point increase in CS, the HR for OS was 3.89 (95% CI, 1.80-8.43). The combined cohort included 248 patients; 126 were male, median (IQR) patient age was 63.3 (53.3-70.3) years, median (IQR) CS was 4 (4-5) points and median (IQR) duration of follow up was 16.6 (9.3-23.5) months. A total of 152 patients had CS less than or equal to 4 points and 96 patients had a CS greater than 4 points. The most common primary tumors represented were small intestinal (n = 136), pancreatic (n = 58), unknown primary (n = 26), and lung (n = 14). With regard to prior surgical or locoregional therapy, 142 patients had undergone primary tumor resection, 73 patients had undergone surgical debulking, and 109 patients had undergone liver-directed therapy. A total of 140 patients received 3 or 4 doses of PRRT, 82 patients received 0 doses, and 26 patients received 1 or 2 doses of PRRT. Among the 82 patients who received a non-PRRT (0 doses) treatment, a majority (n = 57) received everolimus. Baseline patient characteristics of the combined, validation, and original cohorts are detailed in Table 1.

**Table 1.** Comparison of Patient and Tumor Characteristics Among Original, Validation, and Combined Cohorts

Patient or tumor characteristics	Patients, No.			
	Original (N = 122)	Validation (N = 126)	Combined (N = 248)	*P* value
Sex				
Male	62	64	126	>.99^a^
Female	60	62	122	
Median (IQR) age, y	61.8 (53.2-70.1)	63.6 (52.9-70.7)	63.3 (53.1-70.3)	.74^b^
WHO grade				
Grade 1	31	27	58	.03^a^
Grade 2	58	78	136	
Grade 3	12	13	25	
Low grade NOS	21	8	29	
Primary tumor location				
Small intestine	66	70	136	.48^a^
Pancreas	33	25	58	
Lung	8	6	14	
Unknown primary	9	17	26	
Thymus	2	1	3	
Gastric	2	2	4	
Colon	2	5	7	
Median No. (IQR) of prior systemic therapies	1 (1-2)	1 (1-2)	1 (1-2)	.30^b^
PRRT doses received				
0	31	51	82	.03^a^
1 or 2	10	16	26	
3 or 4	81	59	140	
Median CS	5	3	4	<.001^b^
CS				
≤4	59	93	152	<.001^a^
>4	63	33	96	

Abbreviations: CS, Clinical Score; NOS, not otherwise specified; PRRT, peptide receptor radionuclide therapy; WHO, World Health Organization.

^a^ Pearson test.

^b^ Wilcoxon test.

### Outcomes

**Figure 2.**
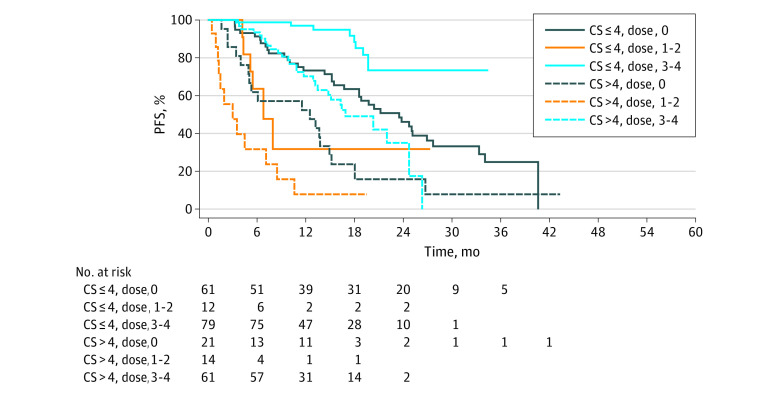
Kaplan-Meier Curves for Progression-Free Survival (PFS) in Patients, Based on Clinical Score (CS) and PRRT Doses Administered PRRT indicates peptide receptor radionuclide therapy.

PFS was estimated by the Kaplan-Meier method. Among patients who received 3 or 4 doses of PRRT, patients with a CS less than or equal to 4 points experienced a median PFS of not reached (NR) (95% CI, NR-NR) whereas patients with a CS greater than 4 points experienced a median PFS of 16.92 months (95% CI, 13.50-24.74 months). Among patients who received 0 doses of PRRT, patients with a CS less than or equal to 4 points experienced a median PFS of 23.52 months (95% CI, 16.76-26.94 months) whereas patients with a CS greater than 4 points experienced a median PFS of 12.55 months (95% CI, 4.99-14.95). Among patients who received 1 or 2 doses of PRRT, patients with a CS less than or equal to 4 points experienced a median PFS of 6.83 months (95% CI, 4.37 months to NR) whereas patients with a CS greater than 4 points experienced a median PFS of 3.06 months (95% CI 1.25-7.16 months) (Figure 2) (*P* < .001, log-rank test). On multivariable Cox regression, adjusting for primary tumor site, tumor grade, and PRRT doses received, for each 2-point increase in CS, the HR for PFS was 2.52 (95% CI, 1.89-3.36). No interaction between PRRT doses administered and CS was observed.

**Figure 3.**
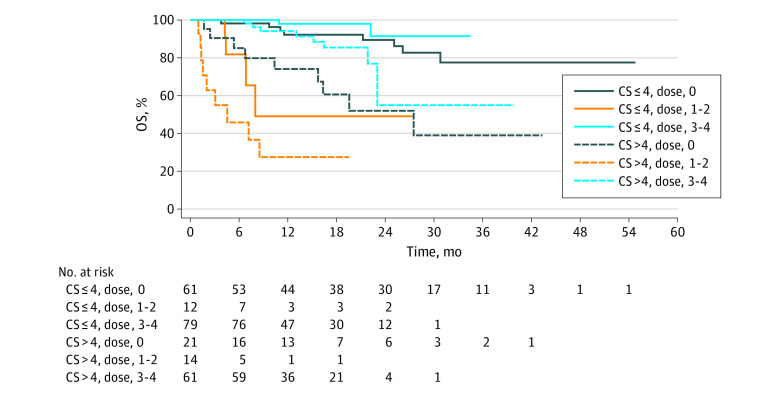
Kaplan-Meier Curves for Overall Survival (OS) in Patients, Based on Clinical Score (CS) and PRRT Doses Administered PRRT indicates peptide receptor radionuclide therapy.

OS was estimated by Kaplan-Meier analysis. Among patients who received 3 or 4 doses of PRRT, patients with a CS less than or equal to 4 points experienced a median OS of NR (95% CI, NR-NR) whereas patients with a CS greater than 4 points experienced a median OS of NR (95% CI, 23 months to NR). Among patients who received 0 doses of PRRT, patients with a CS less than or equal to 4 points experienced a median OS of NR (95% CI, NR-NR) whereas patients with a CS greater than 4 points experienced a median OS of 27.47 months (95% CI, 10.35 points to NR). Among patients who received 1 to 2 doses of PRRT, patients with CS less than or equal to 4 points experienced a median OS of 7.98 months (95% CI, 4.37 months to NR) whereas patients with a CS greater than 4 points experienced a median OS of 4.53 months (95% CI, 1.35 months to NR) (Figure 3) (Log-rank test *P* < .001). On multivariable Cox regression, adjusting for PRRT doses received, for each 2-point increase in CS the HR for OS was 3.48 (95% CI 2.33-5.18).

A total of 31 patients experienced an objective response with PRRT (ORR, 18.7%). No evidence of difference in the proportion of patients who achieved an ORR was observed between patients with CS less than or equal to 4 points or CS greater than > 4 points . Among the patients who received PRRT, 93 (55.4%) experienced symptomatic improvement of pretreatment disease-related symptoms. Patients with CS less than or equal to 4 points were not found to be more likely to derive symptomatic benefit from PRRT compared with patients with CS greater than 4 points (59% [54 patients] vs 52% [39 patients]; *P* = .34; χ^2^ = 0.812).

### Adverse Events

With regard to AEs among the patients who received PRRT, 98 patients developed a total of 168 hematologic AEs, and 60 patients developed a total of 95 nonhematologic AEs. A total of 29 patients experienced grade 3 or higher hematologic AEs, and 13 patients experienced grade 3 or higher nonhematologic AEs (Table 2). No evidence of difference in the development of grade 3/4 treatment–related hematologic AE numbers (participants × events) or grade 3/4 treatment–related nonhematologic AE numbers were observed between patients with CS less than or equal to 4 points or CS greater than 4 points.

**Table 2.** Grade 3 or Higher Hematologic and Nonhematologic Adverse Events Experienced by Patients Who Received ^177^Lu-dotatate

Type of adverse event	Grade 3	Grade 4	Grade 5
Hematologic			
Anemia	7	NA	NA
Lymphopenia	15	NA	NA
Neutropenia	2	NA	NA
Leukopenia	2	NA	NA
Thrombocytopenia	3	NA	NA
Nonhematologic			
Abdominal pain	1	NA	NA
Ascites	1	NA	NA
Bone pain	1	NA	NA
Bilirubin rise	1	1	NA
Diarrhea		1	NA
Hypotension	1	NA	NA
Ileus		1	NA
Small bowel obstruction	1	NA	NA
Vision loss	NA	1	NA
Seizure	1	NA	NA
Transaminase rise	NA	1	NA
Liver failure	NA	NA	1

Abbreviation: NA, not applicable

### Prespecified Subgroup Analyses

PRRT outcomes in select prespecified groups were also reported (eTable in the Supplement). No evidence of difference in PFS (log-rank test *P* = .60) nor OS (log-rank test *P* = .50) was observed between patients who had or had not received prior liver-directed therapy (eFigure 1 in the Supplement).

No evidence of difference in PFS (log-rank test *P* = .70) nor OS (log-rank test *P* = .70) was observed between patients who had or had not undergone prior surgical debulking. Evidence of difference in OS (log-rank test *P* < .001) but not PFS (log-rank test *P* = .20) was observed in patients who had received 3 or 4 doses of PRRT, based upon whether a dose reduction was necessary (eFigure 2 in the Supplement).

## Discussion

The results of this cohort study, which represent the aggregate experience of 4 high-volume NET centers, validate the CS as a tool that can be used to estimate the potential benefit a patient may derive from ^177^Lu-dotatate. The CS is the first score of its kind reported and its simplicity and prospective derivation are strengths that we believe will foster its use among oncologists in the clinic.

After finding that the CS was associated with PFS and OS in in our validation cohort, we combined the validation and original cohorts into a combined cohort for this analysis to improve the estimation and evaluation of the Cox regression models. The most pertinent findings from the analysis pertain to the association of the CS with PFS. By Kaplan-Meier analysis, patients with CS less than or equal to 4 points or greater than 4 points who received 3 or 4 doses of PRRT demonstrated a median PFS of NR (95% CI, NR-NR) and 16.92 months (95% CI, 13.50-24.74 months), respectively. By Kaplan-Meier analysis, patients with a CS less than or equal to 4 points or greater than 4 points who received 1 or 2 doses of PRRT experienced a median PFS of 6.83 and 3.06 months, respectively. For the first time, to our knowledge, we can now provide an estimate of anticipated benefit from ^177^Lu-dotatate for a patient initiating the treatment, based upon CS. On multivariable Cox regression, for each 2-point increase in CS, the HR for PFS increased by 2.52 times. Although it is not surprising that patients with high CS (CS>4) experienced worse outcomes with PRRT than patients with low CS (CS≤4), this result suggests, even among patients with low CS, that increases in the score are associated with poorer PRRT outcomes. Given the contribution of prior treatments and degree of metastatic involvement (disease-related symptoms, tumor bulk in critical organs, peritoneal carcinomatosis) to the CS, this data suggests that earlier incorporation of ^177^Lu-dotatate, when patients are less pretreated and possess a lower degree of metastatic involvement, may lead to better outcomes. We can only comment on the CS score’s ability to estimate given that the association between increased CS and worsening treatment outcomes was also observed in patients receiving a non-PRRT treatment. In the original cohort, it appeared that the CS was associated with PFS only for patients receiving 3 or 4 doses of PRRT and not for those receiving a non-PRRT treatment.^9^ However, given the significant baseline differences between the original and validation cohorts (Table 1), it is unsurprising that the interaction between PRRT doses received and CS was not preserved in the validation or combined cohorts.

Interestingly, we observed the worst treatment outcomes in patients who received 1 or 2 doses of ^177^Lu-dotatate. This finding cannot be explained merely by baseline disease features in this patient group given that CS distribution did not meaningfully vary between patients receiving 1 to 2 doses or 3 to 4 doses of PRRT. Outcomes in patients incompletely treated with PPRT have not been reported before in existing literature. This patient population may need to be further studied through pooled real-world cohort analyses to delineate the reasons for such poor outcomes with ^177^Lu-dotatate; one hypothesis is that a minimum of 3 doses of ^177^Lu-dotatate are needed to achieve a meaningful threshold of DNA damage in NETs to stop tumor growth.

With regard to PRRT outcomes in our prespecified subgroups of interest, we observed no difference in PFS or OS among patients who had or had not undergone prior liver-directed therapy. This is relevant because the question of when to use liver-directed therapy vs PRRT for patients with liver dominant disease and hepatic progression is still debated.^10,11^ Our data suggests that there appears to be no disadvantage to using PRRT after liver-directed therapy, with the caveat that chemoembolization and bland embolization were the predominant types of embolotherapy used. No differences in PFS or OS were observed among patients who had or had not undergone prior surgical debulking; disease bulk at time of PRRT initiation, not necessarily initial disease bulk, appears to influence treatment outcomes for patients.^12^ A decreased OS, but not PFS, was observed among patients who received 3 or 4 doses of ^177^Lu-dotatate with dose reductions compared with those who did not need dose reductions. The OS difference, rather than PFS difference, suggests that perhaps the ability to receive subsequent therapies, rather than PRRT response, could explain this finding. This result warrants prospective evaluation given findings from other series which suggest PRRT dose reductions may limit the effectiveness of the therapy.^13^

### Limitations

Our analysis carries several limitations. First, although the original cohort patients underwent prospective CS assignment, the validation cohort patients underwent retrospective CS assignment. The single investigator from Vanderbilt responsible for assigning CS was blinded to patient outcomes during the time of score assignment to mitigate bias. Second, patients in the analysis possessed a relatively short follow-up period from PRRT or alternative treatment initiation. The short follow-up period resulted in a limited number of death events which decreased the covariates we were able to include in the Cox regression model exploring the relationship between CS and OS. However, PFS was the primary endpoint of the analysis, and a robust Cox regression model found an unequivocal association of CS with PFS. Further, PFS may be a better endpoint than OS to assess the value of a treatment in patients with NETs, given the sheer number of treatments, and sequencing possibilities, available. Third, we cannot comment on the capacity of the CS to specifically predict PRRT response. To truly demonstrate this, a much larger future analysis is necessary. We believe the CS should be tested as a correlative in prospective clinical trials with ^177^Lu-dotatate, to further delineate its prognostic vs predictive utility, given the magnitude of the presented findings.

## Conclusions

This cohort study has found the CS to be the first validated clinical metric, to our knowledge, that can estimate the anticipated benefit from ^177^Lu-dotatate for individual patients. Our study findings suggest that using ^177^Lu-dotatate when patients are less pretreated and possess a lower degree of metastatic involvement may optimize treatment outcomes; this notion, however, requires prospective confirmation from ongoing clinical trials.

## Notes

Supplement. **eTable.** PFS and OS Outcomes in Prespecified Groups Treated with ^177^Lu-dotatate **eFigure 1. **Kaplan-Meier Curves for PFS (A) and OS (B) in Patients Receiving PRRT, Based Upon Whether They Had or Had Not Received Prior Liver-Directed Therapy **eFigure 2. **Kaplan-Meier Curves for PFS (A) and OS (B) in Patients Receiving 3-4 Doses of PRRT, Based Upon Whether They Had or Had Not Required a Dose Reduction
