# Supplementary material for: External Validation of a Clinical Score for Patients With Neuroendocrine Tumors Under Consideration for Peptide Receptor Radionuclide Therapy
Source: JAMA Netw Open. 2022 Jan 19;5(1):e2144170. doi: 10.1001/jamanetworkopen.2021.44170 (PMC8771294; doi:10.1001/jamanetworkopen.2021.44170)
Supplement: Supplement. — eTable. PFS and OS Outcomes in Prespecified Groups Treated with 177Lu-dotatate eFigure 1. Kaplan-Meier Curves for PFS (A) and OS (B) in Patients Receiving PRRT, Based Upon Whether They Had or Had Not Received Prior Liver-Directed Therapy eFigure 2. Kaplan-Meier Curves for PFS (A) and OS (B) in Patients Receiving 3-4 Doses of PRRT, Based Upon Whether They Had or Had Not Required a Dose Reduction [file jamanetwopen-e2144170-s001.pdf]

Supplemental Online Content

Das S, Chauhan A, Du L, et al. External validation of a clinical score for patients with neuroendocrine tumors under consideration for peptide receptor radionuclide therapy. *JAMA Netw Open.* 2022;5(1):e2144170. doi:10.1001/jamanetworkopen.2021.44170

eTable. PFS and OS Outcomes in Prespecified Groups Treated with ^{177}Lu-dotatate

eFigure 1. Kaplan-Meier Curves for PFS (A) and OS (B) in Patients Receiving PRRT, Based Upon Whether They Had or Had Not Received Prior Liver-Directed Therapy

eFigure 2. Kaplan-Meier Curves for PFS (A) and OS (B) in Patients Receiving 3-4 Doses of PRRT, Based Upon Whether They Had or Had Not Required a Dose Reduction

This supplemental material has been provided by the authors to give readers additional information about their work.

© 2022 Das S et al. *JAMA Network Open.*

eTable. PFS and OS Outcomes in Prespecified Groups Treated with ^{177}Lu-dotatate.

Groups	Median PFS (months)	P-value (by Log-rank test)	Median OS (months)	P-value (by Log-rank test)
Peritoneal Carcinomatosis		.2		.8
Yes	NR		NR	
No	22		NR	
Prior Liver-Directed Therapy		.6		.5
Yes	22		NR	
No	24.7		NR	
PRRT Dose Reduction (Among Patients who Received 3-4 Doses of PRRT)		.2		.008
Yes	24.7		NR	
No	26.3		NR	
Liver Dominant Disease Only		.9		.7
Yes	NR		NR	
No	22		NR	
Prior Surgical Debulking		.7		.7
Yes	26.3		NR	
No	24.7		NR	
Primary Tumor Resection		.05		.3
Yes	26.3		NR	
No	17.4		NR	

Abbreviations: NR, not reached; PRRT, peptide receptor radionuclide therapy; OS, overall survival; PFS, progression-free survival

© 2022 Das S et al. *JAMA Network Open.*

A.

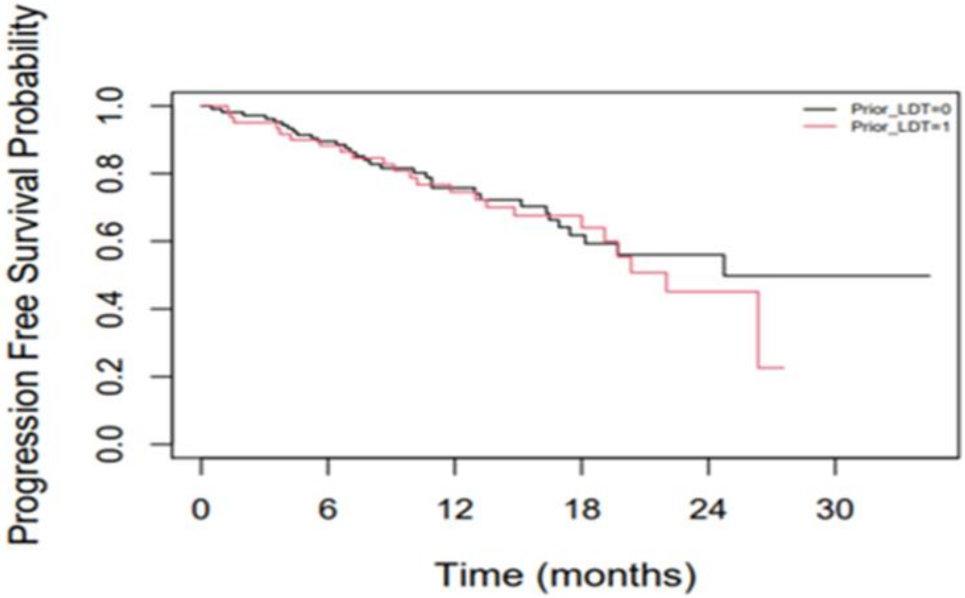

B.

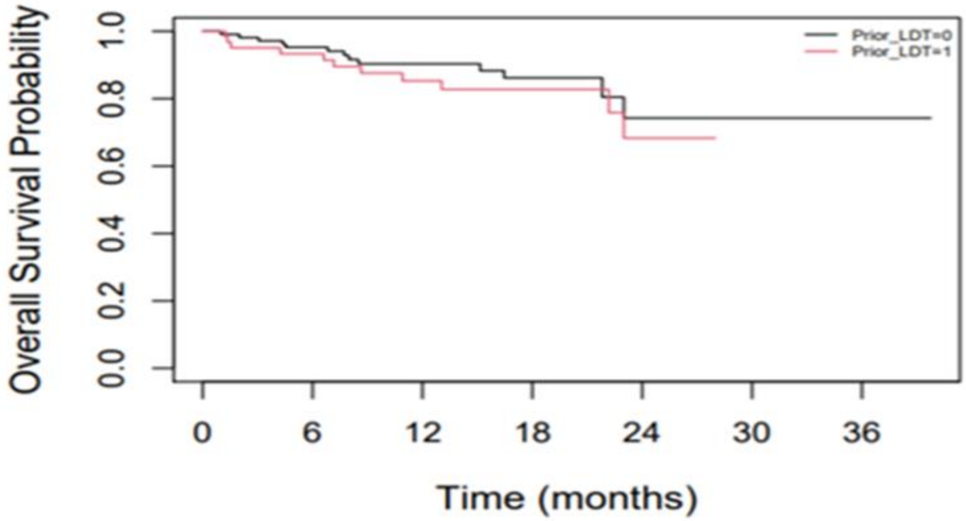

eFigure 1. Kaplan-Meier curves for PFS (A) and OS (B) in patients receiving PRRT, based upon whether they had or had not received prior liver-directed therapy.

Abbreviations: PFS, progression-free survival; OS, overall survival; PRRT, peptide receptor radionuclide therapy; LDT, liver-directed therapy

© 2022 Das S et al. *JAMA Network Open.*

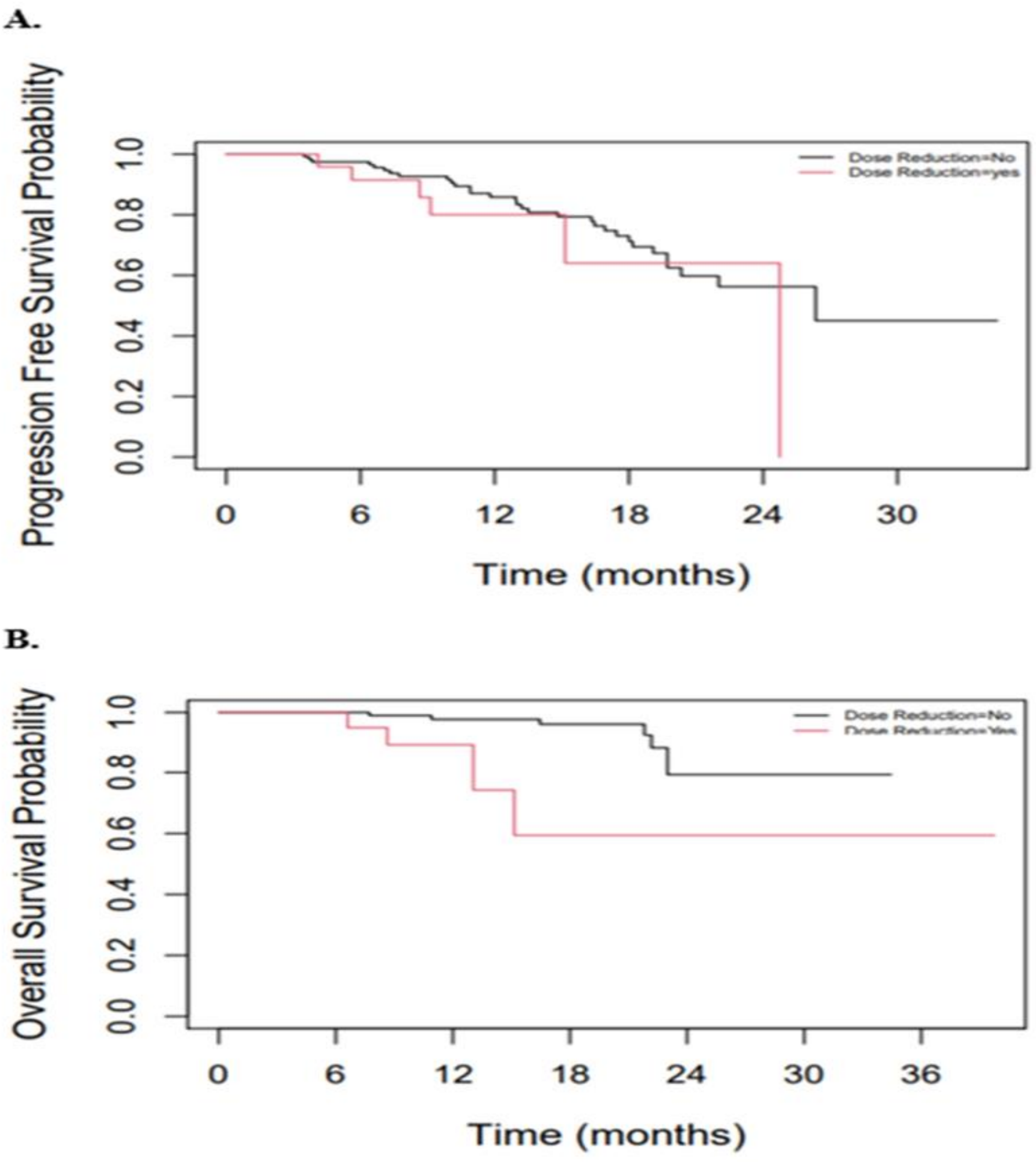

eFigure 2. Kaplan-Meier curves for PFS (A) and OS (B) in patients receiving 3-4 doses of PRRT, based upon whether they had or had not required a dose reduction.

Abbreviations: PFS, progression-free survival; OS, overall survival; PRRT, peptide receptor radionuclide therapy

© 2022 Das S et al. *JAMA Network Open.*
